# Electronic and structural properties of ZnO, Fe₂O₃, and Al₂O₃ clusters interacted with graphene oxide: a combined DFT and experimental study

**DOI:** 10.1038/s41598-026-66145-5

**Published:** 2026-08-17

**Authors:** Ahmed Abdelhady A. Khalil, Mohamed H. Sayed, Asmaa Ibrahim, Hanan Elhaes, Medhat A. Ibrahim

**Affiliations:** 1 Laser Sciences and Interactions Department, National Institute of Laser Enhanced Sciences (NILES), Cairo University, Giza, 12613 Egypt; 2 Solid State Physics Department, National Research Centre, 33 El-Bohouth St., Dokki, Giza, 12622 Egypt; 3 Molecular and Fluorescence Spectroscopy Lab, Centre of Excellence for Advanced Science, National Research Centre, 33 El-Bohouth St., Dokki, Giza, 12622 Egypt; 4 Physics Department, Faculty of Women for Arts, Science and Education, Ain Shams University, Cairo, 11757 Egypt; 5 Spectroscopy Department, National Research Centre, 33 El-Bohouth St., Dokki, Giza, 12622 Egypt; 6 Center for Converging Sciences and Emerging Technologies (CoSET), Benha National University (BNU), El-Obour, 13518 Egypt; 7 Molecular Modeling and Spectroscopy Laboratory, Centre of Excellence for Advanced Science, National Research Centre, 33 El-Bohouth St., Dokki, Giza, 12622 Egypt

**Keywords:** Graphene oxide hybrids, DFT, HOMO/LUMO energy gap, MESP and electronic surface properties, Chemistry, Materials science, Nanoscience and technology, Physics

## Abstract

The work uses Density Functional Theory (DFT) with the B3LYP/LANL2DZ method to study the electronic and structural characteristics of Graphene Oxide (GrO) combined with ZnO, Fe₂O₃, and Al₂O₃. The research examines how GrO anchoring sites, including hydroxyl (-OH), epoxy (-O), and carboxyl (-COOH) groups, affect the performance of these composite materials. The results show that the hydroxyl site creates the largest conductivity improvement because it decreases the energy gap of GrO from 2.7606 eV to 0.3894 eV in the iron oxide hybrid. The Total Dipole Moments (TDM) of molecules with epoxy (O) interactions reach 8.7375 Debye, which indicates that those molecules have greater chemical sensing sensitivity. For studying the surface and stability of the model molecules, both the Molecular Electrostatic Potential, MESP, and QTAIM are calculated. Both quantities are considered complex and site -dependent electronic behavior; applying these calculations can indeed tune the electronic structure by picking specific functional groups. The research demonstrates that the physical integration of metal oxides with graphene oxide results in the formation of a composite material with enhanced properties, as the experimental data (confirmed by spectroscopic and microscopic analyses) align with the theoretical predictions derived from computational modeling. Results dedicated the studied models and/or materials for sensors and electrodes, as well as for catalytic work.

## Introduction

Hybrid nanostructures built on graphene oxide (GrO) have attracted sustained attention because of the chemical versatility imparted by its oxygenated functional groups, which are: carboxyl (-COOH), hydroxyl (-OH), and epoxy oxygen (-O). These moieties not only provide anchoring sites for metal oxides but also redistribute charge density, thereby enabling interfacial engineering at the atomic scale^1,2^. Among candidate oxides, zinc oxide (ZnO) stands out as a direct wide-bandgap semiconductor (3.37 eV) with a high exciton binding energy (60 meV). Its strong optoelectronic and photocatalytic activity makes ZnO an ideal partner for GrO, where synergistic coupling enhances charge separation, tunes band alignment, and improves structural durability^3–7^. Experimental studies further indicate that ZnO incorporation strengthens mechanical and tribological properties of GrO composites^5,6^, yet the precise binding motifs at individual oxygenated sites remain unresolved.

To address this gap, Density Functional Theory (DFT) provides a rigorous quantum-mechanical framework capable of resolving binding energetics, electronic redistribution, and interfacial charge transfer^8–10^.

DFT reveals that graphene act as semiconductors, while graphene oxide behaves as a semi-metal due to the strong polar influence of its carboxyl group. Furthermore, removing the carboxyl group can trigger a transition to semiconductor behavior^11^.

Earlier theoretical work has suggested charge transfer pathways and band-gap modulation in ZnO/GO heterostructures, particularly under doping conditions and with van der Waals corrections^12,13^. However, systematic comparisons across COOH, OH, and O sites are still missing. At the GrO/ZnO interface, subtle orbital hybridization and charge redistribution dictate exciton dissociation, interfacial stability, and transport behavior parameters that ultimately govern photocatalytic and optoelectronic performance. Reports consistently highlight enhanced charge separation and band-gap modulation in ZnO-GO hybrids^12,13^, while DFT analyses emphasize the pivotal role of oxygenated groups in mediating charge transfer and orbital realignment^14,15^. By providing site-specific insights, DFT enables predictive understanding that transcends empirical observation and informs rational material design^9,16^.

Beyond ZnO composites, DFT has been widely applied to molecular systems, yielding descriptors such as HOMO/LUMO energies, total dipole moment, molecular electrostatic potential (MESP), and Quantum Theory of Atoms in Molecules (QTAIM) parameters^17–23^. These descriptors serve as quantitative markers of polarity, reactivity, and stability. For instance, the HOMO/LUMO gap is a benchmark for kinetic stability and chemical reactivity, while MESP mapping provides a visual and numerical framework for identifying electrophilic and nucleophilic regions^24–26^. Together, these parameters establish the foundation for predicting molecular interactions and even biological activity in novel chemical entities^27^.

DFT proves that the electronic properties of ZnO is changed because of doping^28,29^. Magnesium (Mg) doping influences the properties of amorphous ZnO nanoparticles, revealing that Mg atoms preferentially bond with Zn rather than O and tend to locate near the center of the nanoparticle. The doping process effectively modulates the electronic structure, reducing the HOMO-LUMO energy gap to a minimum at 7.5% Mg content before increasing it at higher concentrations, while also significantly shifting the LUMO level to higher energies^28^. Furthermore, higher sulfur (S) levels cause a substantial reduction in the energy gap of ZnO from 4.71 eV to 0.68 eV driven by the upward shifting of the HOMO level and downward shifting of the LUMO level. Ultimately, these modifications enhance the conductivity and reactivity profile of the ZnO nanoparticles^29^.

Aluminum oxide (Al₂O₃) offers another compelling case. Its stable dielectric properties and compatibility with flexible barrier layers make GO/Al₂O₃ composites promising candidates for humidity sensing^30^. The distribution of oxygen functionalities within GO critically influences adsorption dynamics and sensing responses, underscoring the need for atomistic modeling that captures site-specific interactions^31^. Although progress has been made in GO/metal oxide composites for sensing, systematic quantum-mechanical studies remain limited^32^. In this context, DFT provides a robust platform to quantify binding energetics, electronic redistribution, and dielectric permittivity, thereby offering predictive guidance for GO/Al₂O₃ humidity sensors^33,34^. Incorporating Al₂O₃ nanoparticles into GrO frameworks further extends applications to environmental remediation and multifunctional device engineering^35^.

DFT has thus arisen as a foundation of computational materials science, allowing analytical exploration of molecular performance and material properties without the constraints of costly or hazardous experiments^36–39^.

In the present study, ZnO, Fe₂O₃, and Al₂O₃ interactions with three representative oxygenated functional groups of graphene oxide GrO (-COOH, -OH, and -O) are systematically examined at the B3LYP/LANL2DZ level. Key descriptors, including total dipole moment, HOMO/LUMO energy gap, MESP, and QTAIM parameters, are computed to elucidate binding energetics, charge redistribution, and orbital realignment. This comparative, site-resolved analysis clarifies how distinct functionalities modulate interfacial behavior and establishes a mechanistic foundation for tailoring GrO/metal oxide nanocomposites toward enhanced photocatalytic, sensing, and tribological applications. Such precise articulation of objectives ensures that the novelty, scope, and scientific contribution of the manuscript are immediately evident to reviewers.

## Materials and methods

### Details of calculations

**Fig. 1:**
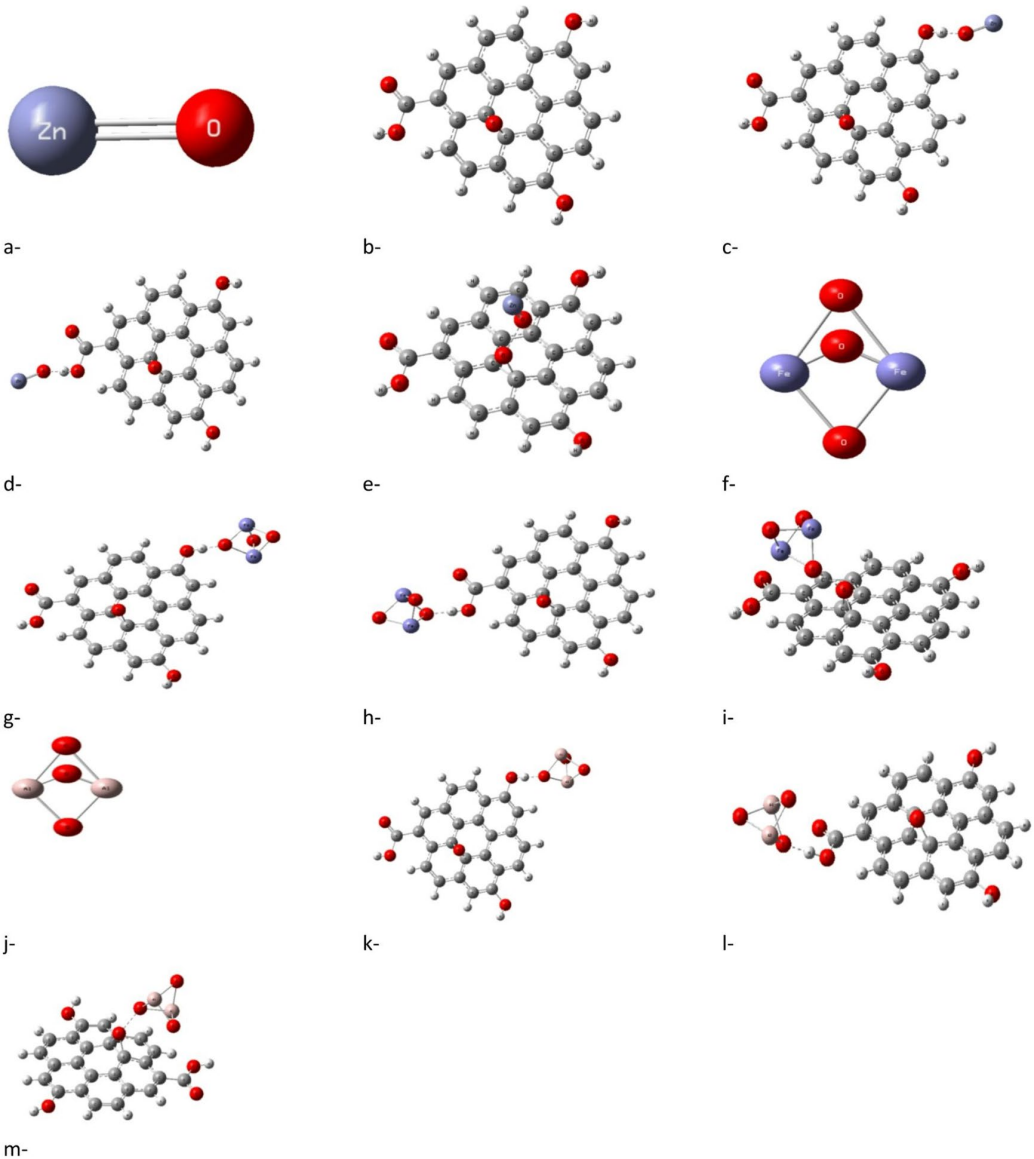
Proposed model structures (**a**) Zinc oxide (ZnO); (**b**) Graphene oxide (GrO); (**c**) ZnO/GrO interacted via OH; (**d**) ZnO/GrO interacted via COOH; (**e**) ZnO/GrO interacted via O; (**f**) Iron oxide (Fe_2_O_3_); (**g**) Fe_2_O_3_/GrO interacted via OH; (**h**) Fe_2_O_3_/GrO interacted via COOH; (**i**) Fe_2_O_3_/GrO interacted via O; (**j**) Aluminium oxide (Al_2_O_3_); (**k**) Al_2_O_3_/GrO interacted via OH; (**l**) Al_2_O_3_/GrO interacted via COOH and (**m**) Al_2_O_3_/GrO interacted via O.

Before discussing the computational details, it is necessary to describe the studied model molecules. Figure 1 presents the proposed model structures used in this study, illustrating how metal oxides (ZnO; Fe_2_O_3_ & Al_2_O_3_) interact with the functional groups of Graphene oxide namely OH; COOH and O. The individual base structures are Zinc oxide (ZnO) as indicated in Fig. 1-a and Graphene oxide (GrO) as shown in Fig. 1-b, along with Iron oxide (Fe_2_O_3_) as indicated in Fig. 1-f. These structures represent ZnO interacting with GrO through different functional sites via the hydroxyl group (OH), as in Fig. 1-c, the carboxyl group (COOH), as in Fig. 1-d, and the epoxy/oxide group (O), as in Fig. 1-e. Similarly, Fe_2_O_3_ is shown interacting with GrO via OH (Fig. 1-g), COOH (Fig. 1-h), and O (Fig. 1-i). Aluminium oxide (Al_2_O_3_), indicated in Fig. 1-i, interacts via OH (Al_2_O_3_/GrO) as indicated in Fig. 1-k. Figure 1-l indicated Al_2_O_3_/GrO interacted via COOH, and finally Fig. 1-m indicated Al_2_O_3_/GrO interacted via O.

The computational investigation of the ZnO and Fe_2_O_3_ with graphene oxide (GrO) hybrid structures was conducted using the Gaussian 09 (G09) software suite^40^ at the Molecular Modeling and Spectroscopy Laboratory, Centre of Excellence for Advanced Science, National Research Centre. All molecular models were optimized and analysed using the B3LYP exchange-correlation functional, which integrates Becke’s three-parameter hybrid functional with the Lee-Yang-Parr correlation functional^41–43^. To really capture the electronic traits of the metallic sites with enough fidelity, the LANL2DZ basis set was adopted. Even so, this kind of approach brings along a fair bit of computational effort, it still ends up giving a balanced treatment between accuracy and efficiency, so it works out.

This basis set effectively utilizes effective core potentials (ECPs) for heavier elements, such as zinc and iron. This specific model chemistry was utilized to calculate key physical and electronic parameters, including total energy, Total Dipole Moment (TDM), and the HOMO-LUMO energy gap (ΔE), as well as to generate Molecular Electrostatic Potential (MESP) maps for each configuration. Global reactivity descriptors are essential computational tools for understanding and predicting the chemical behavior of molecules, calculated primarily from HOMO and LUMO energies using DFT methods, and include parameters such as ionization potential (IE), electronic affinity (**a**), chemical potential (µ), chemical hardness (η), absolute softness (S) and electrophilicity index (ω) were computed with the Eqs^44,45^. :

1 $$\:IP=-{E}_{HOMO}$$

2 $$\:A=-{E}_{LUMO}$$

3 $$\:\mu\:\:=\frac{IP\:+A}{2}$$

4 $$\:\eta\:=\frac{IE-A}{2}$$

5 $$\:S=\frac{1}{\eta\:}$$

6 $$\:{\upomega\:}=\frac{{\mu\:}^{2}}{2\eta\:}$$

To assess the stability of the studied models as well as intermolecular interactions after hydration, the quantum theory of atoms in molecules (QTAIM) using Multiwfn and visual molecular dynamics (VMD) software^46,47^.

### Sampling

For experimental verification of the models, composite materials were prepared.

Graphene oxide (GrO) was prepared using a modified Hummers method as indicated earlier^48^. Zinc Oxide (ZnO); Ferric Oxide (Fe_2_O_3_) and Aluminium oxide (Al_2_O_3_) were purchased from Sigma Aldrich (St. Louis, MO, USA).

Solid phase reaction was followed to prepare composite materials as 500 mg of GrO is mixed with 500 mg separately with ZnO, Fe_2_O_3,_ and Al_2_O_3_.

Each composite material is thoroughly mixed in agate mortar for 6 h, then subjected to Raman, FTIR, UV-Vis, and Confocal microscope imaging to be compared with molecular modeling calculations.

### Instruments

#### Raman spectroscopy measurement

Raman measurements were performed using a confocal Raman microscope (WiTec Alpha 300 RA, made in Germany) with 532 nm excitation, and 30 s integration with 10 accumulations at a single point.

#### Fourier Transform Infrared Spectroscopy (FTIR) spectroscopy measurements

FTIR spectra were acquired with a Bruker Vertex 70 FTIR spectrometer (Bruker Optik GmbH, Germany), utilizing the Potassium Bromide (KBr) pellet technique. Spectra were recorded across the mid-infrared region of 4000 –400 cm⁻¹ with a resolution of 4 cm⁻¹. Each spectrum represents an average of 50 scans to enhance the signal-to-noise ratio.

#### UV-Vis spectroscopy measurements

The optical diffuse reflectance spectra (DRS) were measured by (Jasco V570) UV- VIS-NIR spectrophotometer.

## Results and discussions

### Interaction of zinc oxide (ZnO) with graphene oxide (GrO)

Table 1 presents the calculated electronic properties, including total dipole moment TDM, highest occupied molecular orbital HOMO, lowest unoccupied molecular orbital LUMO, and energy gap ΔE for the studied structures. As indicated in Table 1, the interaction between ZnO and GrO significantly alters the electronic properties of the composite material, as evidenced by the TDM and energy gap (ΔE) values.

The TDM reflects the reactivity and polarity of the structures. GrO has a TDM of 4.7427 Debye, while that of ZnO was 5.5504 Debye. When ZnO interacts via the O the TDM was 4.2126 Debye. When ZnO interacts via the COOH group, the TDM goes up to 5.9236 Debye, so it seems like the system is more reactive. On the other hand, if the interaction happens through the OH group, the TDM drops down to 2.3869 Debye, which lines up with the radical shift in its energy gap.

Table 1 shows that pristine GrO has an energy gap of around 2.7606 eV, and then when ZnO is added, the electronic behavior doesn’t really do one single thing. Depending on where ZnO sits, the response changes a bit. The strongest modulation shows up near the hydroxyl group, so ZnO adsorption there pulls the gap down to roughly 0.6672 eV, which basically points to a big boost in the surface conductivity. By comparison, when the interaction happens through the carboxyl site, written as -COOH, or through the epoxy oxygen –O, the gaps come out to about 2.7108 eV and 2.7951 eV. Those values stay fairly close to unmodified GrO. So, overall, the picture looks like the OH location strongly disturbs the electronic arrangement, while the COOH and O locations keep a rather stable electronic profile.

Based on HOMO/LUMO energies some reactivity descriptors were calculated. Table 2 shows the computed global reactivity descriptors: chemical hardness (η), softness (S), electronegativity (χ), chemical potential (µ), and electrophilicity index (ω). These were obtained from the HOMO and LUMO energy levels that are listed in Table 1. Altogether, these quantities form a sort of numerical scaffold that can be used to judge stability, chemical reactivity, and charge‑transfer tendency in oxide/graphene oxide (GrO) composites. Basically, this table summarizes the main electronic traits that decide how the hybrid frameworks act on the chemical side, even if the specific details are not spelled out line by line.

The ionization potential (IP) and electron affinity (EA) tell you the energy required to pull out an electron, and the energy released (or gained) when an electron is added, respectively. When the IP is high (ZnO at 6.6287 eV), it usually means the electronic configuration is more stable, and therefore less prone to react. Meanwhile, higher EA values (Fe_2_O_3_/GrO (OH) at 4.9833 eV) point toward a stronger willingness to accept electrons.

Chemical hardness (η) measures resistance to change in electron distribution. Chemical softness (S) functions as the opposite value of hardness. ZnO/GrO (OH) and Fe_2_O_3_/GrO (OH) exhibit extremely low hardness (0.3336 eV and 0.1947 eV) and high softness (2.9975 eV^− 1^ and 5.1361 eV^− 1^).

The specific composites demonstrate high reactivity because their elevated softness level indicates their tendency to form covalent bonds and transfer charges.

Electronegativity (χ) and Chemical Potential (µ): Electronegativity describes the power of the structure to attract electrons. The chemical potential represents the electron escape tendency from the system through its negative value of electronegativity. The Electrophilicity Index (ω) measures how much energy decreases when the maximum electron transfer occurs between a donor and an acceptor.

Each metal oxide is supposed to interact with GrO from three sites; results indicated that the type of functionalization and/or coordination on the GrO surface significantly alters the reactivity of the metal oxide composite.

Hydroxyl functionalization dramatically increases the reactivity. The Fe_2_O_3_**/**GrO (OH) has the highest electrophilicity index (68.8531 eV) and the highest softness in the set. This suggests that the OH group facilitates much easier electronic transitions compared to the (COOH) or (O) groups. Composite structures with epoxide (O) and carboxyl (COOH) groups generally maintain higher chemical hardness. The global reactivity descriptors show some kind of different stability and reactivity patterns across the composites that were studied. Like, the ZnO/GrO (O) case, it has a hardness of 1.3976 eV, so it looks more chemically steady than the hydroxyl‑functionalized one. When you compare the pure oxides it’s clear that ZnO is “harder” (1.1883 eV) than Fe₂O₃ (0.7463 eV) and that points to Fe₂O₃ being inherently more reactive, sort of yeah. After integrating with GrO, the electrophilicity index (ω) generally goes up which suggests stronger electron‑accepting ability. Pure ZnO starts at ω = 12.4537 eV but then it jumps to 34.4036 eV when it’s bonded at the hydroxyl site of GrO, that jump is significant, and it signals a strong inclination for electron uptake.

Looking across the composites, ZnO/GrO (O) comes out as the most chemically stable arrangement, because it has the top hardness value (1.3976 eV). On the other hand, Fe₂O₃/GrO (OH) is reported as the most reactive and the least stable, with the lowest hardness 0.1947 eV and the largest softness 5.1361 eV⁻¹. And yes, the same Fe₂O₃/GrO (OH) composite has the highest electrophilicity index (68.8531 eV) which really supports its pronounced electron‑accepting behavior.

Meanwhile, pristine GrO shows the weakest electron affinity, with ω = 5.8328 eV. Then if softness is treated like a reactivity kind of yardstick, ZnO/GrO (O) stays the least reactive structure, with softness of 0.7155 eV⁻¹.

**Fig. 2:**
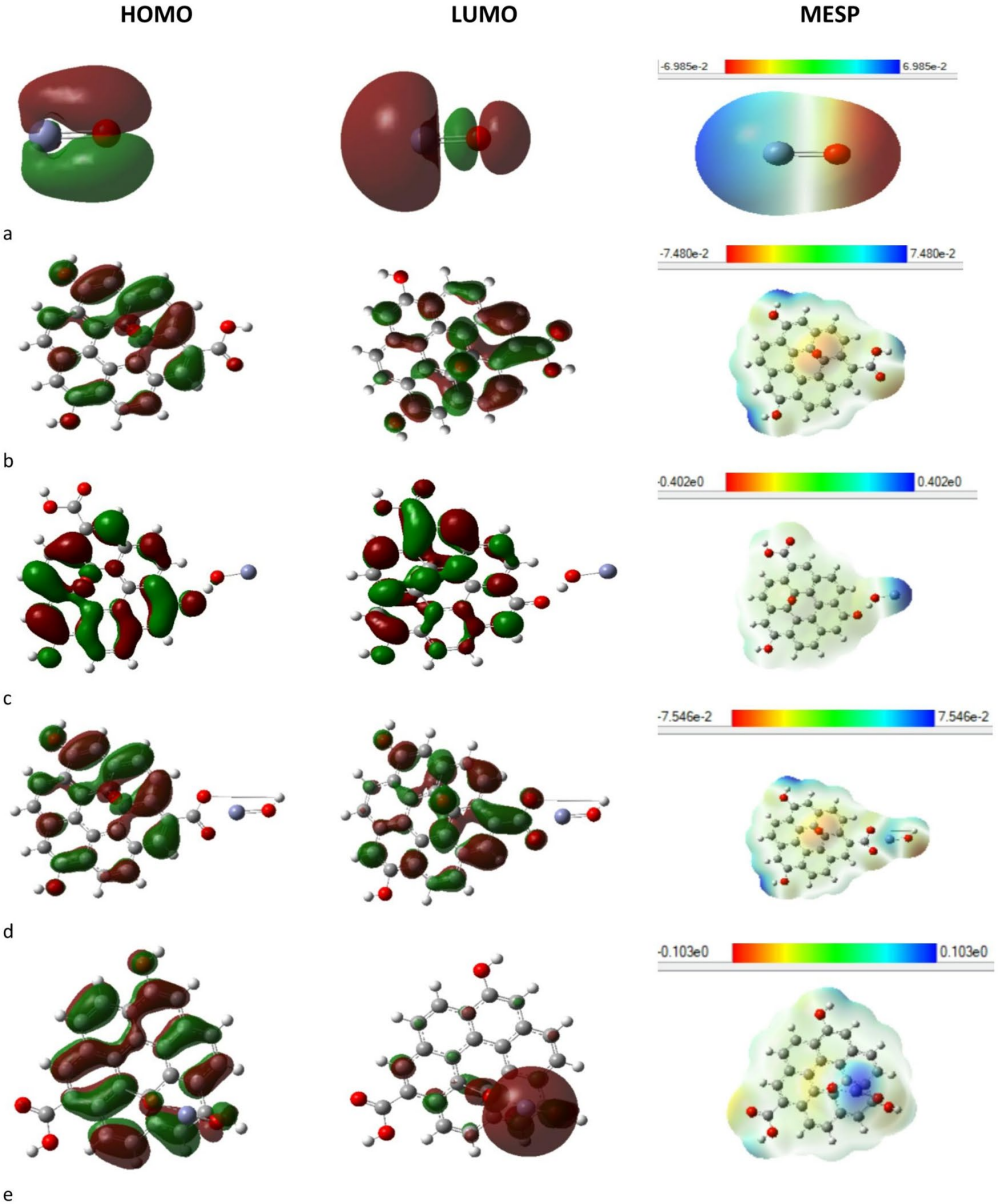

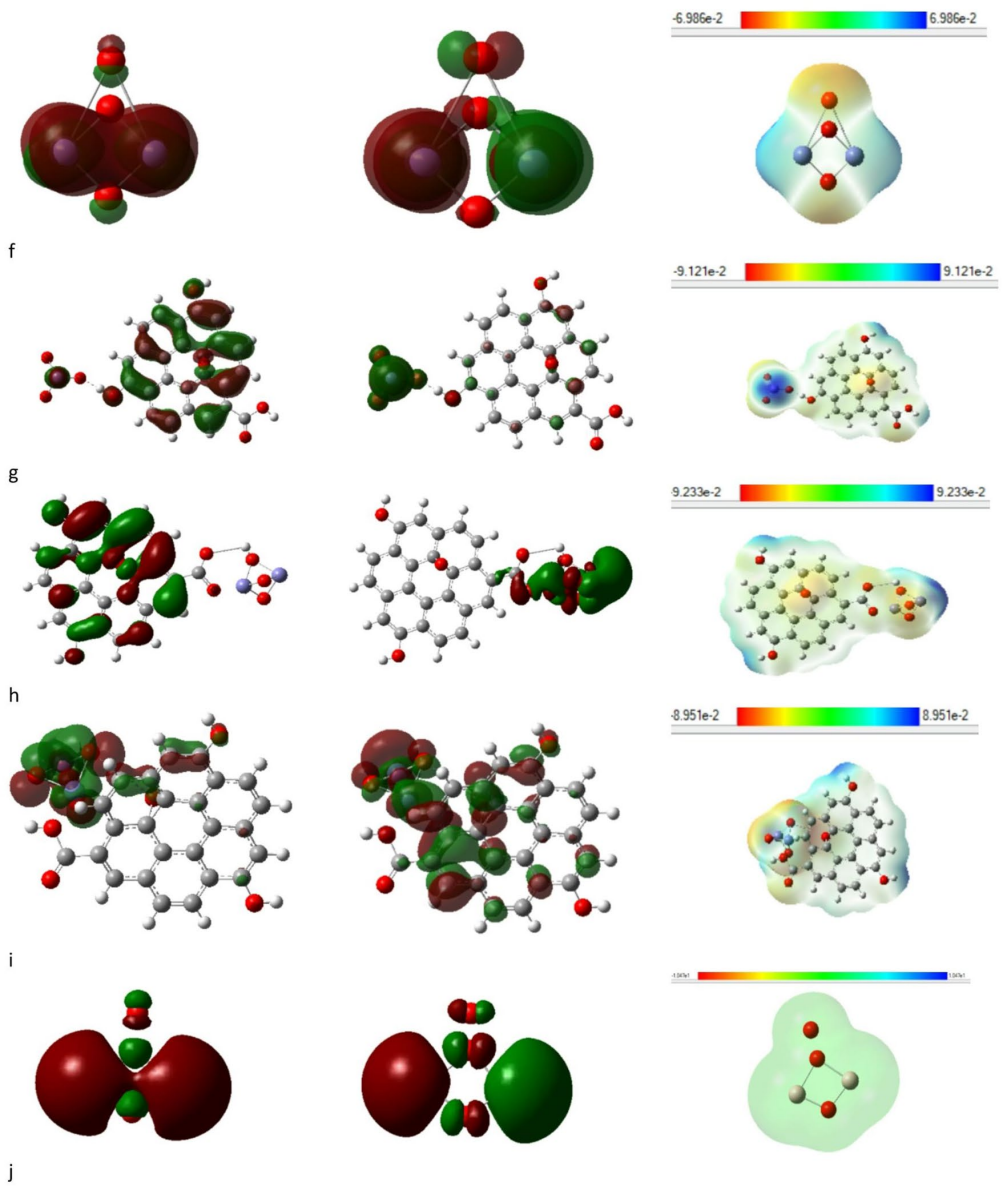

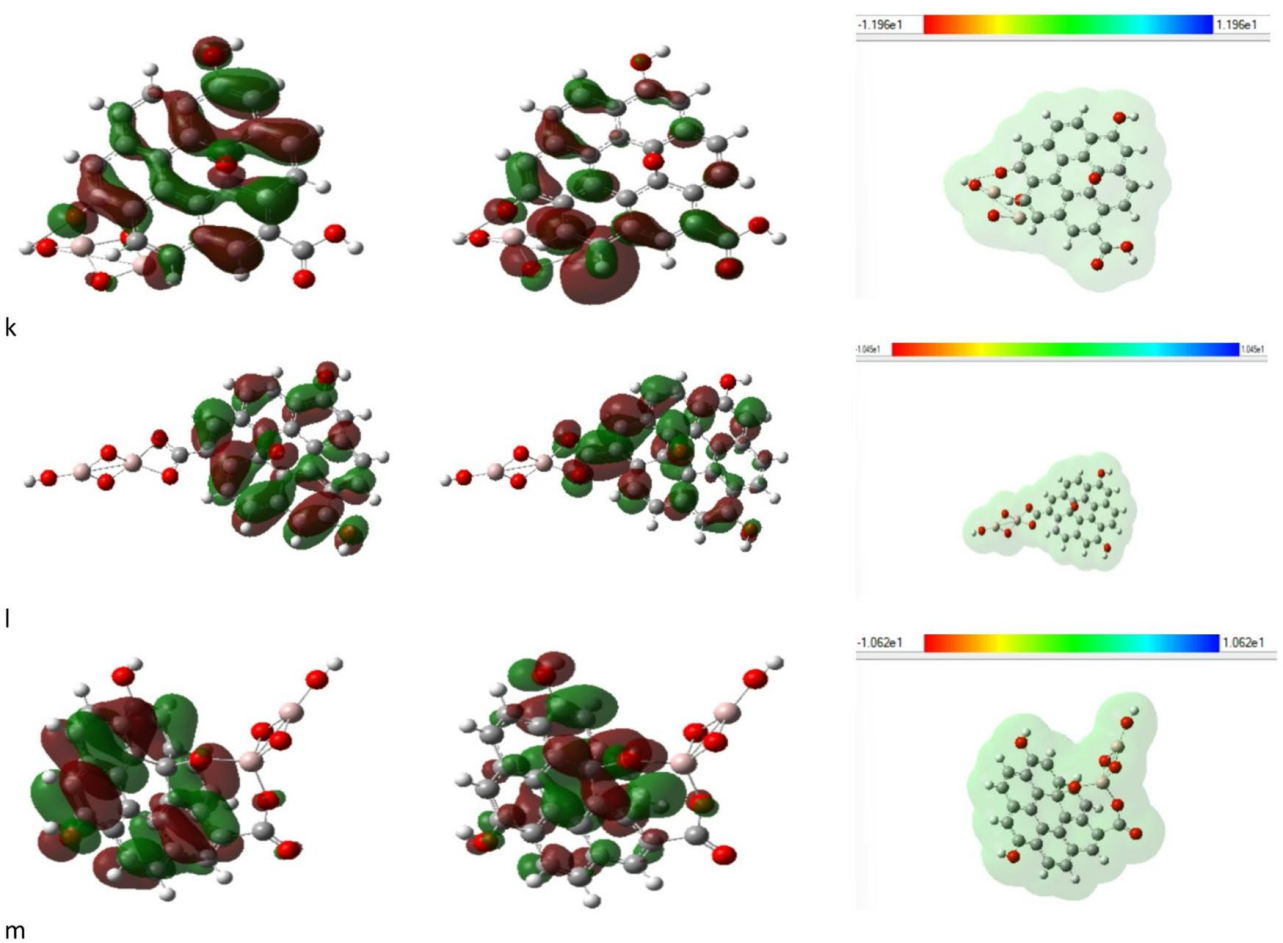
HOMO; LUMO and MESP for the studied structures whereas (**a**) Zinc oxide (ZnO); (**b**) Graphene oxide (GrO); (**c**) ZnO/GrO interacted via OH; (**d**) ZnO/GrO interacted via COOH; (**e**) ZnO/GrO interacted via O; (**f**) Iron oxide (Fe_2_O_3_); (**g**) Fe_2_O_3_/GrO interacted via OH; (**h**) Fe_2_O_3_/GrO interacted via COOH; (**i**) Fe_2_O_3_/GrO interacted via O; (**j**) Aluminium oxide (Al_2_O_3_); (**k**) Al_2_O_3_/GrO interacted via OH; (**l**) Al_2_O_3_/GrO interacted via COOH and (**m**) Al_2_O_3_/GrO interacted via O.

Figure 2 shows a picture of the electronic states, with the HOMO, LUMO, and MESP distributions included. The HOMO/LUMO images are used to show how the electron density is spread out over space, and for the ZnO/GrO hybrids (c, d and e) a clear change in charge distribution is observed right along the interface, between the oxide domains and the graphene functional groups. That kind of interfacial rearrangement, in other words, supports the idea that an integrated electronic system is formed, and not just some simple physical placement of separate parts together.

The Molecular Electrostatic Potential (MESP) maps, instead, show how the charge distribution shifts from place to place, and they also point out the regions of higher versus lower electron density that are induced once ZnO is attached. In the ZnO/GrO hybrids (Fig. 2c, d and e), you can see this MESP redistribution more clearly when compared to pure GrO and pure ZnO. Put a bit differently, the ZnO cluster changes the local potential of the graphene sheet, especially near the bonding sites.

If we look at the interaction site in terms of MESP, as far as ZnO is tied in through OH, the MESP for this case mirrors the dramatic change that also appears in the energy gap (0.6672 eV). In other words, the interface becomes strongly polarized, and that kind of polarization helps conductivity. Now, when ZnO interacts via COOH and O, the MESP images show a more confined or localized charge distribution around those functional groups. That localization matches the observation of higher energy gaps and a more robust, or relatively stable, electronic behavior compared to the version bonded through OH.

Overall, the MESP analysis supports the idea that when ZnO attaches to GrO, it produces a highly polar surface. By visualizing these potentials, we can also see how ZnO acts like a tuning knob for the reactivity of graphene oxide, making the hybrids appropriate for sensors or catalysis, where the surface charge needs to be specific enough to interact with target molecules.

**Table 1:** Calculated electronic properties, including total dipole moment TDM, highest occupied molecular orbital HOMO, lowest unoccupied molecular orbital LUMO, and energy gap ΔE for the studied structures.

Structure	TDM, Debye	HOMO, eV	LUMO, eV	ΔE, eV
ZnO	5.5504	-6.62874576	-4.252084616	2.3767
GrO	4.7427	-5.393067004	-2.632450184	2.7606
ZnO/GrO (OH)	2.3869	-5.124760628	-4.457532196	0.6672
ZnO/GrO (COOH)	5.9236	-5.448034436	-2.737214844	2.7108
ZnO/GrO (O)	4.2126	-5.677428224	-2.882252672	2.7951
Fe_2_O_3_	1.4758	-5.53892118	-4.04636492	1.4926
Fe_2_O_3_/GrO (OH)	4.8026	-5.372658304	-4.983260308	0.3894
Fe_2_O_3_/GrO (COOH)	3.9821	-5.366671752	-4.192491212	1.1742
Fe_2_O_3_/GrO (O)	8.7375	-5.601235744	-3.343489292	2.2577
Al_2_O_3_	1.9563	-6.144107164	-4.70488564	1.4392
Al_2_O_3_/GrO (OH)	9.5594	-5.97430678	-3.8164269	2.1579
Al_2_O_3_/GrO (COOH)	7.5347	-5.676611876	-3.13477632	2.5418
Al_2_O_3_/GrO (O)	14.5049	-5.811853528	-4.38650992	1.4253

**Table 2:** Reactivity descriptors for the studied structures whereas Ionization potential (IP); Electron affinity (EA); Chemical hardness (η); Chemical softness (S); Electronegativity (χ); Chemical potential (µ) and Electrophilicity index (ω).

Structure	IP (eV)	EA (eV)	η (eV)	S (eV⁻¹)	χ (eV)	µ (eV)	ω (eV)
ZnO	6.6287	4.2521	1.1883	0.8415	5.4404	-5.4404	12.4537
GrO	5.3931	2.6325	1.3803	0.7245	4.0128	-4.0128	5.8328
ZnO/GrO (OH)	5.1248	4.4575	0.3336	2.9975	4.7911	-4.7911	34.4036
ZnO/GrO (COOH)	5.4480	2.7372	1.3554	0.7378	4.0926	-4.0926	6.1788
ZnO/GrO (O)	5.6774	2.8823	1.3976	0.7155	4.2798	-4.2798	6.5531
Fe_2_O_3_	5.5389	4.0464	0.7463	1.3400	4.7926	-4.7926	15.3893
Fe_2_O_3_/GrO (OH)	5.3727	4.9833	0.1947	5.1361	5.1780	-5.1780	68.8531
Fe_2_O_3_/GrO (COOH)	5.3667	4.1925	0.5871	1.7033	4.7796	-4.7796	19.4556
Fe_2_O_3_/GrO (O)	5.6012	3.3435	1.1289	0.8858	4.4724	-4.4724	8.8593
Al_2_O_3_	6.1441	4.7049	0.7196	1.3896	5.4245	-5.4245	20.4452
Al_2_O_3_/GrO (OH)	5.9743	3.8164	1.0789	0.9268	4.8954	-4.8954	11.1056
Al_2_O_3_/GrO (COOH)	5.6766	3.1348	1.2709	0.7868	4.4057	-4.4057	7.6363
Al_2_O_3_/GrO (O)	5.8119	4.3865	0.7127	1.4032	5.0992	-5.0992	18.2424

### Interaction of iron oxide (Fe_2_O_3_) with graphene oxide (GrO)

As indicated in Table 1, compared to pure GrO (TDM = 4.7427 Debye) and pure Fe_2_O_3_ (TDM = 1.4758 Debye), the TDM increases slightly to 4.8026 Debye as Fe_2_O_3_ with GrO via Hydroxyl (OH). Then, the TDM decreases to 3.9821 Debye as Fe_2_O_3_ with GrO via Carboxyl (COOH). As the interaction took place via Epoxy (O) this site produces the most significant change, with the TDM rising sharply to 8.7375 Debye, indicating a highly reactive and polar structure.

Regarding HOMO/LUMO Energy Gap (ΔE), the interaction of Fe_2_O_3_ with GrO, the ΔE is drastically reduced to 0.3894 eV, suggesting a massive increase in conductivity corresponding to Fe_2_O_3_/GrO(OH). The gap is lowered to 1.1742 eV corresponding to Fe_2_O_3_/ GrO(COOH).

The Fe_2_O_3_/ GrO(O) interaction results in an energy gap of 2.2577 eV, which is the most electronically stable among the iron oxide hybrids but still lower than pure GrO.

The Molecular Electrostatic Potential (MESP) map analysis for the interaction of Fe_2_O_3_ with GrO is indicated in Fig. 2-f, -g, - h and -i. The maps indicated how the Fe_2_O_3_ modifies the electronic landscape of Graphene Oxide. These maps reveal that the attachment of the metal oxide cluster causes a significant redistribution of charge density across the graphene surface, creating localized regions of high and low potential that are absent in the pure components. Specifically, Fe_2_O_3_ interaction through hydroxyl (-OH) groups gives this super polarized MESP profile, and that lines up directly with the hybrid’s more noticeably reduced energy gap of 0.3894 eV, plus the predicted high conductivity. Meanwhile, when bonding is handled by carboxyl (-COOH) and epoxy (-O) groups you end up with charge distributions that are more localized around the binding sites, so the electronic features stay comparatively steady, relative to that OH-linked setup. Overall, the MESP readout seems to confirm that Fe₂O₃ works like an electronic modulator, shaping a polar, reactive surface, and that’s what helps these hybrids feel more suitable for advanced work like chemical sensing and catalysis.

### Aluminium oxide (Al_2_O_3_) interacted with graphene oxide (GrO)

The TDM for Al_2_O_3_ is 1.9563 Debye, while GrO has a TDM of 4.7427 Debye and an energy gap of 2.7606 eV, shown in Table 1. After hybridization with Al₂O₃, these values then get noticeably altered, depending on the group (-OH, -O, or -COOH). In Fig. 2, the space arrangement of the molecular orbitals and the charge shifting is laid out quite clearly across the hybrid structures. Especially in Fig. 2 the frontier orbitals suggest a kind of localized orbital mixing near the oxygenated sites, not really a uniform redistribution over the whole sheet. The MESP maps also go on to show that oxide attachment leads to strong electron density reorganization. You can see electronegative zones gathering more tightly around the oxygen centres of the functional groups, while electropositive regions appear around the aluminium atoms. And this polarization is backed by numbers as well: the TDM increases a lot, going up to 9.5594 Debye for Al₂O₃/GrO (OH) and  7.5347 Debye for Al₂O₃/GrO (COOH) then became14.5049 Debye for Al₂O₃/GrO (O) . That basically points to these surfaces becoming more responsive for sensing uses.

Table 1 also shows that isolated Al₂O₃ has an energy gap of   1.4392 eV, and a TDM value of 1.9563 Debye. Then when you anchor onto GrO functional groups, the gap gets way smaller, so the whole system ends up in a semiconductor like regime. The hydroxyl linked Al₂O₃/GrO (OH) layout gives a gap of 2.1579 eV, plus high TDM 9.5594 Debye. This sort of points to it having theability for charge redistribution, and even hints at better conductivity. In comparison, the epoxy (Al₂O₃/GrO (O)) and carboxyl (Al_2_O_3_/GrO (COOH)) setups end up with gaps: 1.4253 eV and  2.5418 eV, respectively. Overall, this trend supports that all three oxygenated environments do activate the insulating oxide, but the epoxy really stands out for electronic excitation and surface polarization.

Looking at reactivity a bit like this, Al₂O₃ turns out to be chemically hard, in the sense that it resists electron rearrangement, you know, it’s not very willing to change. When you bring GrO into the mix, the hybridization with GrO pushes the system toward something softer and more reactive in profile. Pretty clear trends show up: Al₂O₃/GrO (OH) is the one with the smallest chemical hardness η, while also having the largest global softness S. That matches its narrow energy gap and a stronger polarizability overall. Meanwhile, the -COOH and -O anchored setups sit somewhere in the middle; they carry intermediate hardness, which hints at higher electronic stability. Also, the electrophilicity indices ω are elevated for all Al₂O₃/GrO hybrids, and that points to a strong electron‑accepting character. So, in the end, these hybrids look like promising platforms, not only for chemical sensing, but also for targeted molecular interactions, in a more practical, sort of application-minded way.

### Density of states (DOS) analyses

**Fig. 3:**
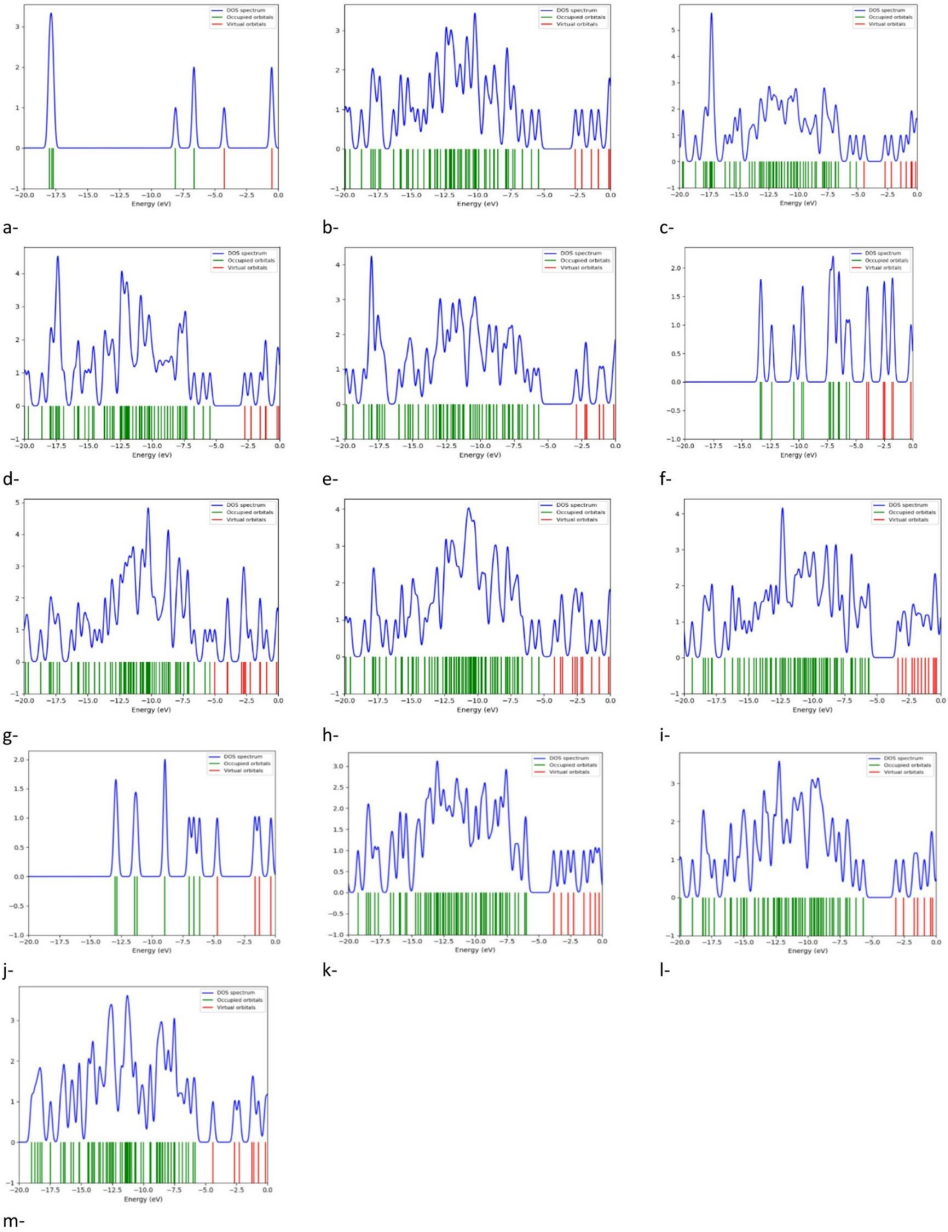
DOS for the studied structures (**a**) Zinc oxide (ZnO); (**b**) Graphene oxide (GrO); (**c**) ZnO/GrO interacted via OH; (**d**) ZnO/GrO interacted via COOH; (**e**) ZnO/GrO interacted via O; (**f**) Iron oxide (Fe_2_O_3_); (**g**) Fe_2_O_3_/GrO interacted via OH; (**h**) Fe_2_O_3_/GrO interacted via COOH; (**i**) Fe_2_O_3_/GrO interacted via O; (**j**) Aluminium oxide (Al_2_O_3_); (**k**) Al_2_O_3_/GrO interacted via OH; (**l**) Al_2_O_3_/GrO interacted via COOH and (**m**) Al_2_O_3_/GrO interacted via O.

Figure 3 presents the Density of States (DOS) and, which gives a kind of direct look at how the electronic levels are distributed versus the HOMO/LUMO energy gaps (ΔE) listed in Table 1. In the DOS plots, you can see that the bonding between metal oxides and graphene oxide (GrO) functional groups tunes the electronic landscape of these composites well. The overall shape and topology of the DOS, change a lot when you consider the anchoring site (-OH, -COOH, or -O). This basically points to the fact that the electronic modulation is quite site-specific, like it depends on where the attachment happens, rather than being fully uniform.

**Fig. 4:**
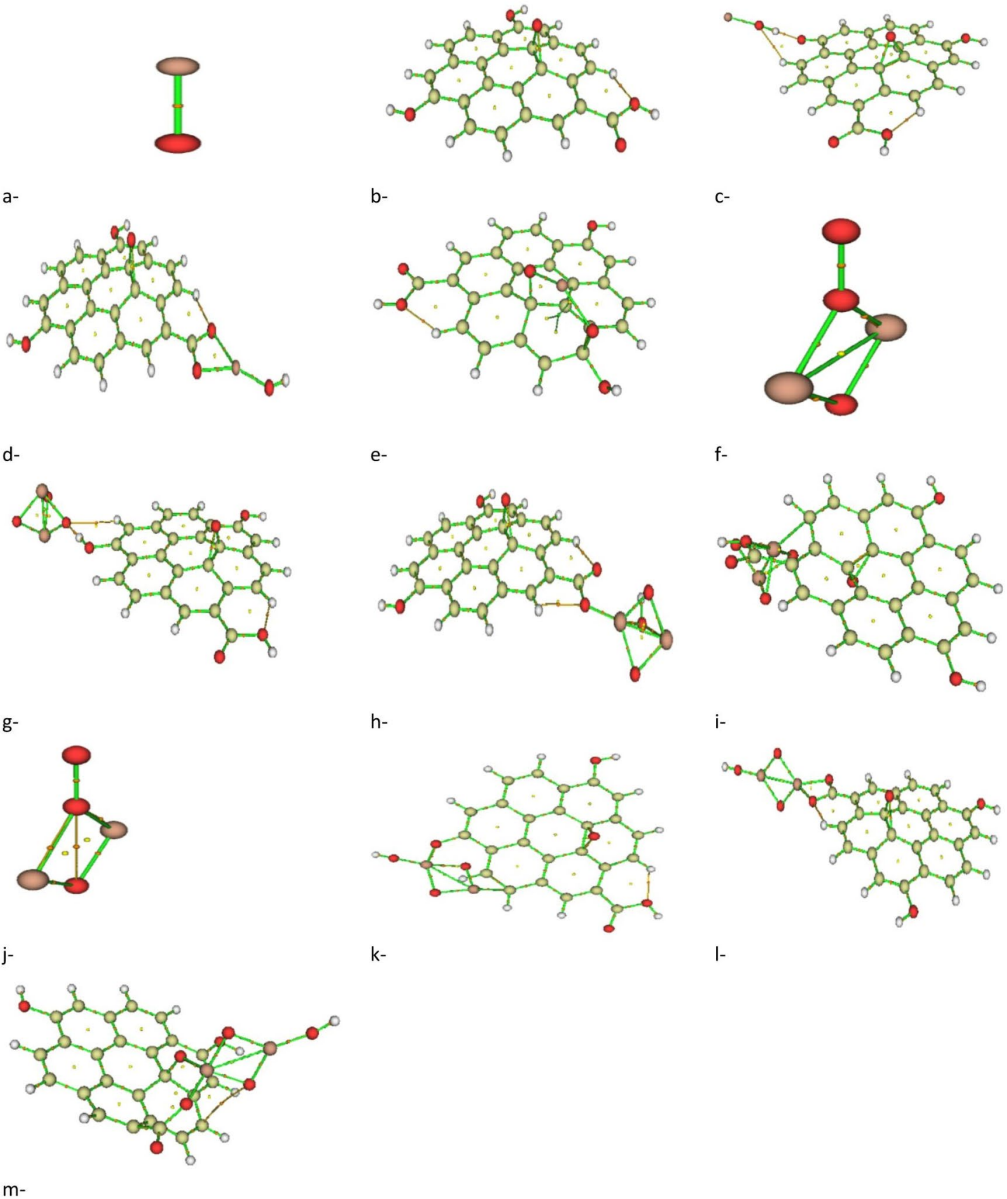
QTAIM topology for the studied structures (**a**) Zinc oxide (ZnO); (**b**) Graphene oxide (GrO); (**c**) ZnO/GrO interacted via OH; (**d**) ZnO/GrO interacted via COOH; (**e**) ZnO/GrO interacted via O; (**f**) Iron oxide (Fe_2_O_3_); (**g**) Fe_2_O_3_/GrO interacted via OH; (**h**) Fe_2_O_3_/GrO interacted via COOH; (**i**) Fe_2_O_3_/GrO interacted via O; (**j**) Aluminium oxide (Al_2_O_3_); (**k**) Al_2_O_3_/GrO interacted via OH; (**l**) Al_2_O_3_/GrO interacted via COOH and (**m**) Al_2_O_3_/GrO interacted via O.

For ZnO/GrO (Fig. 4-c) and Fe_2_O_3_/GrO (Fig. 4-g), the DOS curves show this pretty strong narrowing of the gap between states that are occupied and those that are unoccupied. You can really see the “closing” kind of effect, and it suggests a more integrated electronic system, like the electrons are more connected, which goes along with higher conductivity. Meanwhile, when we look at the interactions driven by carboxyl (-COOH) and epoxy (-O) groups (Figs. 3-d, -e, -h, and -i), they seem to lead to more localized states. Those localized states keep a wider separation, and that ends up indicating better electronic stability, especially when you compare it with the hydroxyl-bonded variants.

These DOS-based observations line up, quantitatively, with the HOMO/LUMO gaps that are listed in Table 1. For pristine GrO, the gap is 2.7606 eV, and then it shifts once oxide attachment happens. The most notable drop shows up at hydroxyl sites, where ΔE drops all the way to 0.6672 eV for ZnO/GrO and to 0.3894 eV for Fe₂O₃/GrO. Those reduced gaps match what is seen in Figs. 3-c and -g, namely denser electronic states, so you can say the surface conductivity increases a lot. In contrast, carboxyl and epoxy interactions tend to hold onto wider gaps. For instance, ZnO/GrO (O) gives 2.7951 eV, and that fits with the DOS shapes that look more separated, and the states appear more localized too. So overall, hydroxyl bonding seems to favour conductive pathways, while epoxy and carboxyl bonding keep the electronic situation steadier and closer to pristine GrO.

The DOS curves in Fig. 3 also make it clear that spatial hybridization reworks how the electronic distribution sits inside the composites. Take Al₂O₃/GrO (OH): Fig. 3(k) shows a dense integration and, at the same time, a visible shift of states toward the Fermi level. That kind of “gap closing” lines up with the smallest calculated ΔE, which is 1.4253 eV in Table 1. This points to the epoxy arrangement as the most effective way to increase conductivity and surface polarization, even if it’s a bit subtle in the middle of the curves.

Meanwhile, bonding through the hydroxyl and carboxyl networks (Fig. 3-l and -m) produces more separated and localized electronic states. Those isolated states explain why the electronic stability stays higher, and the band gaps remain wider, specifically 2.1579 eV and 2.5418 eV, respectively, which are also the values listed in Table 1.

### Quantum theory of atoms in molecules (QTAIM) topography

The Quantum Theory of Atoms in Molecules (QTAIM) analysis, as represented in Fig. 4, provides a topological map of the electron density to characterize the bonding nature of the hybridized structures. This analysis identifies Bond Critical Points (BCPs) and paths that define the stability and strength of the interactions between the metal oxides and graphene oxide (GrO).

Figure 4-a shows a simple topological path between Zn and O, defining the baseline electronic distribution for the metal oxide. Then Fig. 4- b displays the complex network of BCPs within the carbon lattice and its attached oxygen-containing functional groups. Figure 4-f illustrates the triangular/bridged topology of the iron and oxygen atoms within the cluster.

The topology changes based on the bonding site, confirming the “tuning” of electronic properties as indicated in Fig. 4-c. This structure shows a distinct bond path at the hydroxyl site. The QTAIM map supports the drastic reduction in the energy gap to 0.6672 eV, reflecting a highly integrated electronic system with increased conductivity.

Figure 4-d shows localized bond paths at the carboxyl group. The topology indicates a stable interaction that maintains a higher energy gap (2.7108 eV) compared to the OH site.

As the interaction happened via O, as shown in Fig. 4-d, the BCPs here represent the attachment to the epoxy site, resulting in the most electronically stable ZnO hybrid with an energy gap of 2.7951 eV. As iron oxide interacted with GrO, more complex topologies took place due to the multi-atomic nature of the iron oxide. The topology in Fig. 4–g reveals a highly polarized interface. This correlates with the massive increase in conductivity and the lowest energy gap observed in the study (0.3894 eV).

Figure 4 h shows bond paths between the iron cluster and the carboxyl group, resulting in an intermediate energy gap of 1.1742 eV. Finally, Fig. 4-i produces a sharp rise in the TDM (8.7375 Debye). The QTAIM topology confirms a highly reactive and polar structure, though it remains the most stable iron hybrid with a gap of 2.2577 eV.

For ZnO/GrO interactions, it was reported earlier that, besides experimental findings, the DFT results provide a “molecular-level proof” that the resulting oxygen-deficient structure is responsible for the improved performance^49^, while for Fe_2_O_3_/GrO interactions, combining laboratory experiments with DFT provides a comprehensive look at how these materials interact at the atomic level. It is reported that Fe_2_O_3_ increased the potential of the GrO composite for electronic device applications^50^.

The structural rearrangement upon anchoring the multi-centered Al_2_O_3_ cluster onto cyclic frameworks (especially via the carboxyl or bridged epoxy structures) leads to the detection of Ring Critical Points (RCPs) with a (3, + 1) signature.

The geometric mapping for these rather stable RCP loops comes through and kind of, yes, confirms that the Al_2_O_3_/GrO composites stay topologically stable against chemical cleavage, or outright disintegration. This outcome further validates the mechanical robustness and structural resilience of aluminium oxide clusters, reinforcing their suitability for electronic device modeling, moisture‑barrier applications, and humidity‑sensing platforms.

### Experimental verification

To verify the studied model, the real samples were subjected to Raman, FTIR, and UV-Vis spectroscopy as follows.

#### Raman study

**Fig. 5:**
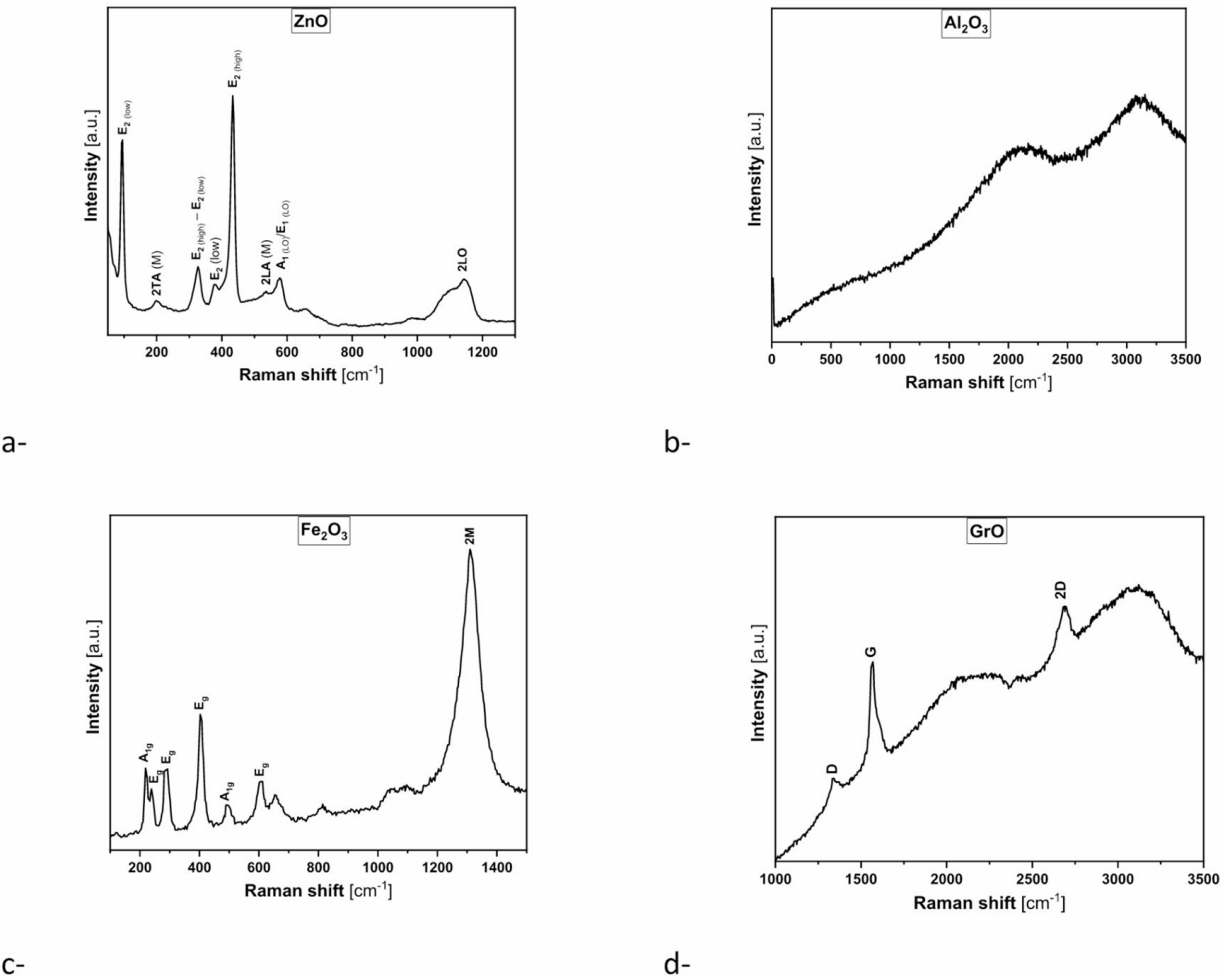
Raman spectra of (**a**) ZnO; (**b**) Al_2_O_3_; (**c**) Fe_2_O_3_, and (**d**) GrO.

The Raman spectra of ZnO, Fe₂O₃, Al₂O₃, graphene oxide (GrO), and their corresponding composites provide clear evidence of their structural characteristics. The pristine ZnO (Fig. 5-a) exhibits well-defined Raman-active modes characteristic of the wurtzite structure, with the most prominent peak located at ~ 437 cm⁻¹ (E₂ high mode), confirming its high crystallinity^51^.

Al₂O₃ does not show any detectable Raman peak under the experimental conditions used, as seen in Fig. 5-b. This missing Raman response is often linked to its wide band gap and a small Raman scattering cross section under 532 nm excitation, so the phonon modes can be kind of hard to catch, especially when the material is in powder form. It looks like this behaviour fits earlier reports too, where alumina frequently gives either extremely weak or practically undetectable Raman signals, unless the measurement is done with optimized settings or higher sensitivity, or both.

Figure 5-c shows a typical hematite Fe₂O₃ fingerprint, with multiple Raman bands appearing in the low- and mid-wavenumber regions (225–613 cm⁻¹), along with the distinct two-magnon scattering feature around ~ 1320 cm⁻¹, which reflects its magnetic ordering and crystalline nature^52^.

Graphene oxide, shown in Fig. 5-d, displays its typical D and G bands at ~ 1350 cm⁻¹ and ~ 1590 cm⁻¹, respectively, confirming the presence of a defective sp² carbon network disrupted by oxygen-containing functional groups^53^.

**Fig. 6:**
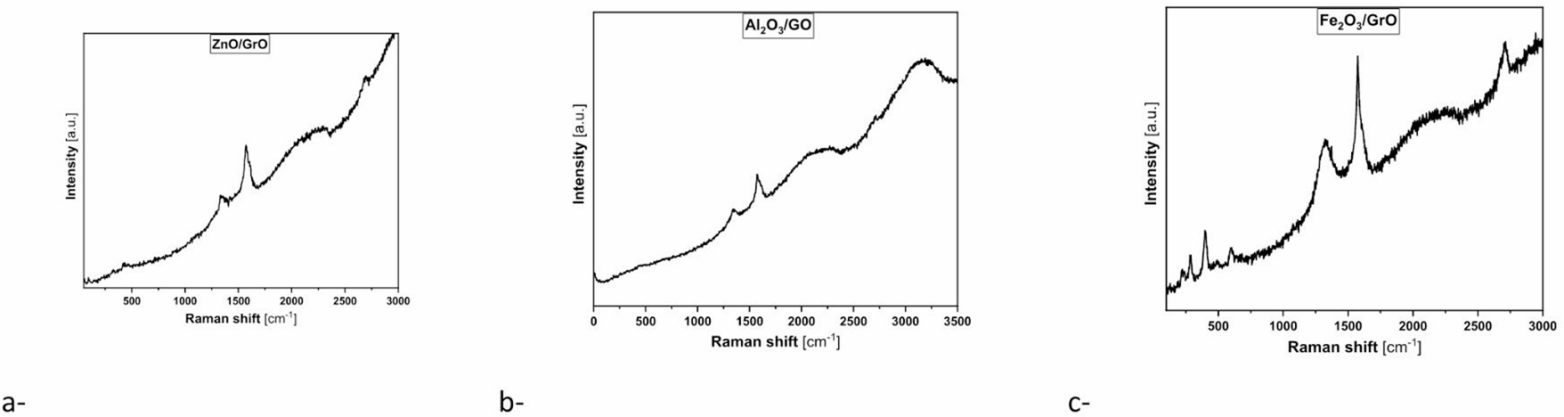
Raman spectra of (**a**) ZnO/GrO; (**b**) Al_2_O_3_/GrO, and (**c**) Fe_2_O_3_/GrO.

In the composite systems (ZnO/GrO, Fe₂O₃/GrO, and Al₂O₃/GrO), the Raman spectra (Fig. 6) show a simple superposition of the individual components. The characteristic D and G bands of GrO remain clearly visible, while the oxide signatures are either preserved (as in ZnO and Fe₂O₃) or remain undetectable in the case of Al₂O₃. Importantly, no new Raman bands, peak shifts, or noticeable broadening are observed, indicating that no chemical bonding or structural transformation occurs during the mixing process. It is also important to note that lower laser power was intentionally used for the composite measurements to avoid laser-induced heating or reduction of GrO. As a result, the Raman intensity of ZnO and Fe₂O₃-related signals appears reduced in the composites; however, this decrease is purely instrumental and does not reflect any loss of crystallinity or phase alteration. Overall, the Raman results strongly support that the composites are physically mixed systems in which each component retains its original structural identity.

#### FTIR study

**Fig. 7:**
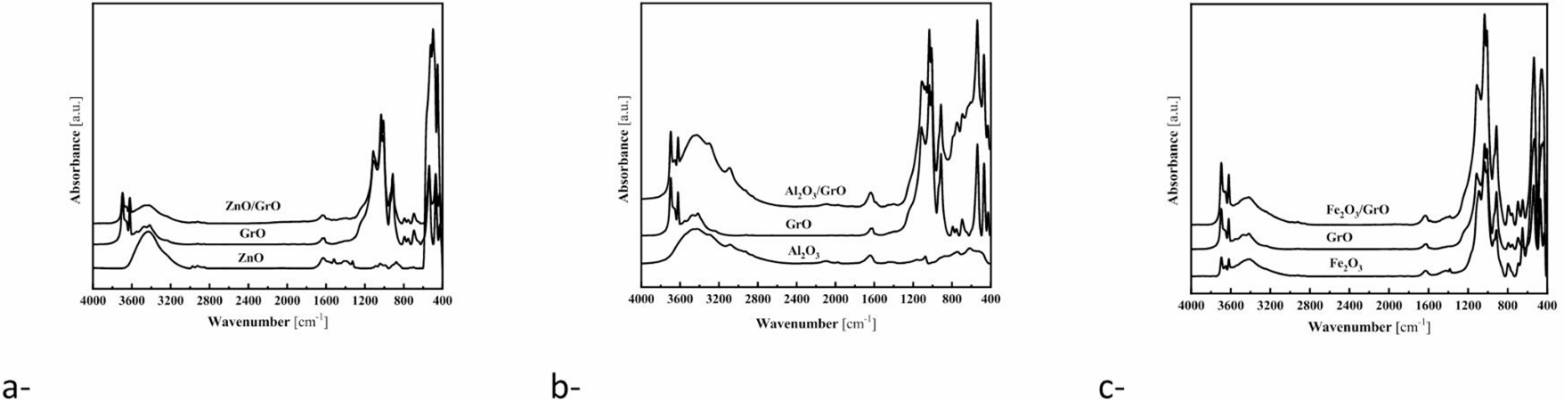
FTIR spectra of pristine graphene oxide (GrO), the metal oxide nanoparticles (ZnO, Al₂O₃ and Fe₂O₃), and their related GrO nanocomposites (ZnO/GrO, Al₂O₃/GrO, and Fe₂O₃/GrO).

The FTIR spectra of the investigated materials are presented in Fig. 7, including pristine graphene oxide (GrO), metal oxides (ZnO, Al₂O₃, and Fe₂O₃), and their corresponding nanocomposites. As shown in Fig. 7a, the FTIR spectrum of GrO exhibits a broad band around 3400 cm⁻¹ corresponding to O–H stretching vibrations, confirming the presence of hydroxyl groups on the graphene surface. Additional peaks at ~ 1720 cm⁻¹, ~ 1620 cm⁻¹, and 1050–1250 cm⁻¹ are attributed to C = O, C = C skeletal vibrations, and C–O stretching modes, respectively.

In Fig. 7b and c, ZnO and Al₂O₃ exhibit characteristic metal–oxygen vibrational bands in the low-wavenumber region (< 800 cm⁻¹), confirming their structural formation. Similarly, Fe₂O₃ (not shown in the first three panels but included in the full figure set) displays its typical Fe–O stretching vibrations in the same region.

Upon formation of the nanocomposites (ZnO/GrO, Al₂O₃/GrO, and Fe₂O₃/GrO), as illustrated in the corresponding spectra, noticeable shifts and intensity variations are observed in the oxygen-containing functional group regions. These changes indicate strong interfacial interactions between GrO and the metal oxide nanoparticles, mainly through hydrogen bonding and electrostatic interactions.

Overall, the spectral modifications observed in Fig. 7 confirm the successful integration of metal oxides within the graphene oxide matrix and highlight the formation of strong interfacial coupling, which is expected to enhance the electronic and surface properties of the prepared nanocomposites.

#### UV-Vis study

The optical properties of ZnO, Al₂O₃, Fe₂O₃, GrO, and their composites were investigated using diffuse reflectance spectroscopy and analyzed through the Kubelka–Munk method combined with Tauc plots. This approach allows reliable estimation of the optical band gaps of powder materials by converting reflectance data into an equivalent absorption coefficient.

**Fig. 8:**
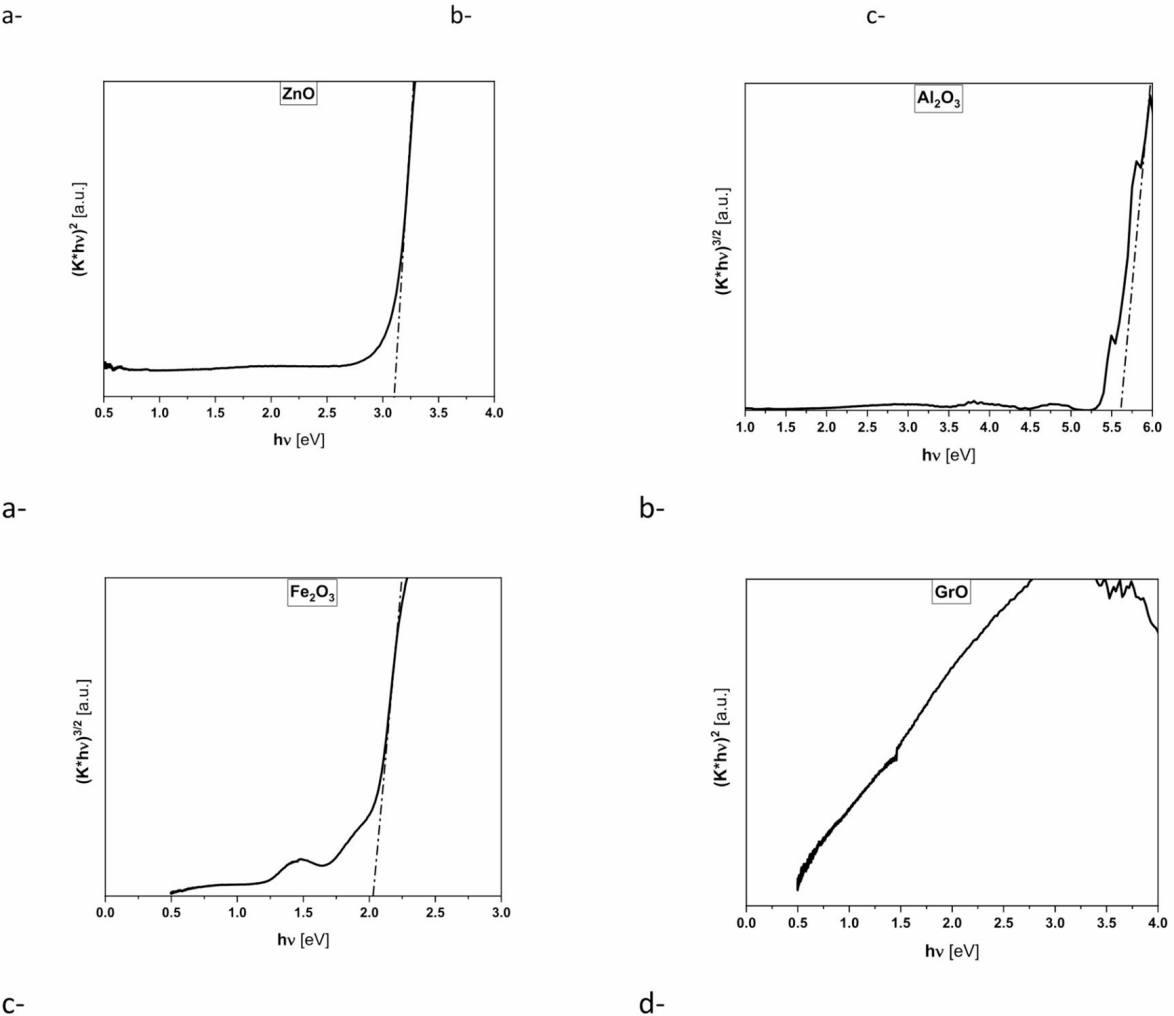
Tauc plots of (**a**) ZnO; (**b**) Al_2_O_3_; (**c**) Fe_2_O_3_, and (**d**) GrO.

ZnO, as shown in Fig. 8-a, exhibits a sharp absorption edge in the UV region, with a calculated direct band gap in the range of 3.1 eV, consistent with its well-known semiconducting nature. Al₂O₃ is a wide-bandgap insulating material, with no significant absorption in the UV-visible region. The estimated band gap (Fig. 8-b) lies in the range of 5.5–6.5 eV, which is lower than the ideal bulk value but reasonable for diffuse reflectance-based estimation, where instrumental limitations and defect states may influence the apparent optical edge. This confirms its insulating nature and electronic stability.

Fe₂O₃ shows strong absorption extending into the visible region, yielding an optical band gap of approximately 2 eV, as shown in Fig. 8-c, which reflects its narrow-band-gap semiconductor behaviour.

Graphene oxide shows a more complex optical response because its electronic structure is disordered. When the sp² carbon network gets disrupted by oxygen functional groups, you end up with localized states, which then makes an apparent band gap that usually lands somewhere between 2.0 and 4.5 eV. The observed range is governed by the oxidation degree and the extent of structural disorder within the system.

**Fig. 9:**
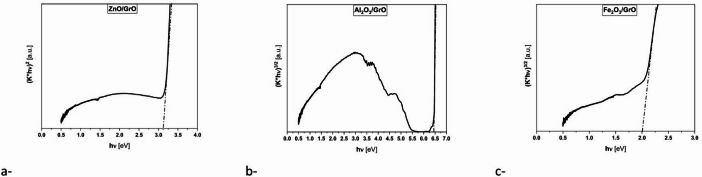
Tauc plots of (**a**) ZnO/GrO; (**b**) Al_2_O_3_/GrO and (**c**) Fe_2_O_3_/GrO.

For the composite materials, the Kubelka-Munk/Tauc plots, illustrated in Fig. 9, show no evidence of new absorption edges or additional electronic transitions. Instead, the optical response of each composite can be interpreted as a combination of the individual components. Any slight variations in the extracted band gap values arise from optical scattering effects, overlap of absorption features, and the heterogeneous nature of powder mixing rather than from true electronic interaction or band structure modification. This observation confirms that the soft mixing approach preserves the intrinsic optical properties of each constituent without inducing chemical bonding or charge-transfer-induced band gap narrowing.

#### Surface study

**Fig. 10:**
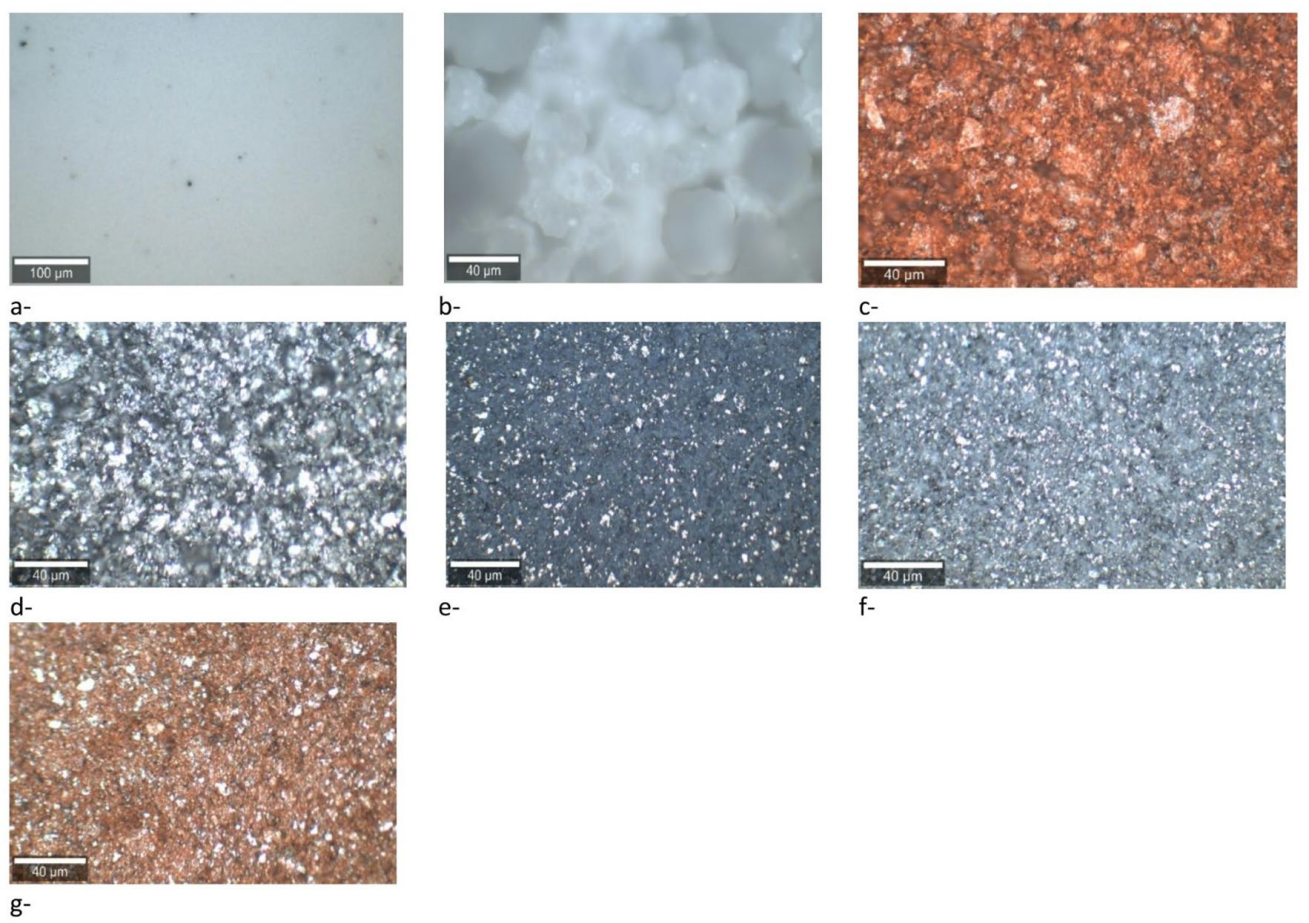
Optical confocal microscopy images for the studied samples (**a**) ZnO; (**b**) Al_2_O_3_; (**c**) Fe_2_O_3_, (**d**) GrO, (**e**) ZnO/GrO; (**f**) Al_2_O_3_/GrO, and (**g**) Fe_2_O_3_/GrO.

Another important description for the prepared composite is to describe its surface with the help of optical confocal microscopy as indicated in Fig. 10.

These microscopy images serve as experimental evidence of the materials’ surface morphology and distribution.

These confocal images offer a visual baseline of the physical samples, confirming their successful preparation and the structural integrity of the synthesized composites.

These images provide the experimental proof to support the Raman, FTIR and molecular models, confirming the physical existence and surface characteristics of the composites discussed throughout the research.

The images demonstrated that each studied metal oxide is integrated into the graphene oxide matrix, which is crucial for the synergistic effects such as enhanced conductivity and sensing performance, which is already predicted by the studied molecular modeling.

It is worth to mention that, the optical microscopy results strictly as complementary surface-morphology observations, while relying on the combined spectroscopic evidence (Raman and FTIR) and the DFT modeling framework to discuss hybrid interactions of the studied cluster systems.

### Dispersion corrections

The studied structures were optimized with B3LYP/LANL2DZ, the model is not account for long-range van der Waals (dispersion) forces. So that, dispersion correction were conducted by adding an empirical dispersion correction via the route section # opt rb3lyp/lanl2dz iop(3/124 = 3) geom=connectivity. Table 3 presented the calculated total dipole moment TDM, and energy gap ΔE for ZnO; GrO; ZnO/GrO (OH) and ZnO/GrO (COOH) before and after dispersion correction. These structures were chosen as an example for the effect of dispersion correction upon the HOMO/LUMO energies and TDM of the studied molecular systems. Results indicated comparable results for one of the studied systems. Results in Table 3 indicated that, for smaller cluster systems yielded results compared to those obtained without them.

**Table 3:** Calculated electronic properties, including total dipole moment TDM, and energy gap ΔE for the studied structures before and after dispersion correction.

Structure	TDM, Debye		ΔE, eV	
	Before	After	Before	After
ZnO	5.5504	5.5504	2.3767	2.3766
GrO	4.7427	4.7137	2.7606	2.7636
ZnO/GrO (OH)	2.3869	2.2464	0.6672	0.6631
ZnO/GrO (COOH)	5.9236	5.9120	2.7108	2.7095

## Conclusion

B3LYP/LANL2DZ calculated data indicated that the obtained models provide definitive evidence, based on which the anchoring position of metal oxides on graphene oxide (GrO) critically dictates the electronic, structural, and chemical behavior of the resulting hybrid nanostructures. Hydroxyl (-OH) functionalization comes out as the most effective “conduit” for conductivity, taking the intrinsic energy gap of pristine GrO (2.7606 eV) down to as low as 0.3894 eV in the Fe₂O₃/GrO (OH) composite. Meanwhile, epoxy (-O) and carboxyl (-COOH) linkages keep wider band gaps, so the electronic stability stays more guarded. Polarization analyses also suggest that epoxy and carboxyl anchoring generate strong dipole moments, and it’s exemplified by 8.7375 Debye in Fe₂O₃/GrO (O), while hydroxyl bonding improves chemical softness (5.1361 eV⁻¹), favouring reactivity.

QTAIM descriptors confirm that –OH connections promote polarizability, whereas -O and -COOH sites retain higher hardness and structural resilience. These findings set up a clear framework for targeted material design. Hydroxyl‑anchored hybrids are best suited for high‑conductivity electronic applications, and epoxy plus carboxyl configurations show advantages for ultra‑sensitive chemical sensing as well as site‑specific catalytic functions. Overall, by showing how anchoring position governs electronic tuning, polarization, and reactivity, this study lays a more mechanistic foundation for rational engineering of metal oxide/GrO nanocomposites toward advanced optoelectronic, sensing, and catalytic technologies.

As the composites interact physically, the samples were prepared experimentally through solid-phase reaction to confirm the physical nature of their interactions.

Raman, FTIR, and confocal microscopy images provide experimental proof to support the physical existence and surface characteristics of the composites discussed throughout the research. Furthermore, gathering spectroscopic, imaging, and modeling data indicated that metal oxides are integrated into the graphene oxide matrix, which is crucial for the synergistic effects such as enhanced conductivity and sensing performance.

Grimme’s dispersion model indicated that for smaller cluster systems yielded results compared to those obtained without them.

## Data Availability

The data supporting the findings of this study can be obtained from the corresponding author upon request, subject to reasonable conditions.
